# Remember S.E.P.A. flap?

**DOI:** 10.4103/0970-0358.63949

**Published:** 2010

**Authors:** A. D. Dias

**Affiliations:** Prof. Emeritus, Department of Plastic Surgery, Lokmanya Tilak Municipal General Hospital (L.T.M.G.H), Sion, Mumbai, India

Sir,

It seems that the authors were completely oblivious of the landmark paper which was published by Dr. A. D. Dias on the "SEPA" flap.

1. The 1^st^ paper was published in the British Journal of Plastic Surgery 1984;37:256-61 as "The Superficial External Pudendal Artery (SEPA) axial pattern flap". In this paper, the anatomy of the flap was studied in 12 cadavers and the flap has was clinically in six patients. A bilateral SEPA flap was also described. Moreover, the anatomical position of the flap makes it possible to immobilize the hand in a much more comfortable and natural position than that assumed in a groin flap. As the flap can be rotated to almost 180 degrees on either side, it is very suitable for defects in the genital region. Thus, it can be used for skin cover in the second stage repair of hypospadius, or a urinary fistula, or in difficult repairs in a hypospadic cripple. It can also add bulk to a hypoplastic penis and its use in epispadias repair is very convenient. The flap is also useful for degloving injuries of the penis and scrotum, in reconstruction of an amputated penis and in the correction of certain contractures in the groin.
2. A second paper: "The Anatomical basis of the SEPA flap" by U. A. Patil, A. D. Dias and R. L. Thatte was published in the British Journal of Plastic Surgery 1987;40:342-7.
3. A third paper: "The use of the SEPA flap in the repair of defects in the hands and fingers" by A. D. Dias, R. L. Thatte, U. A. Patil, L. D. Dhami, S. Prasad was also published in the British Journal of Plastic Surgery 1987;40:348-59.

I hope that I have placed the subject in the proper perspective.

## References

[1] Selvan SS, Alagu GS, Gunasekaran R (2009). Use of a hypo gastric flap and split thickness skin grafting for a degloving inquiry of the penis and scrotum: a different approach. Indian J Plast Surg.

